# From imaging to intervention: emerging potential of PET biomarkers to shape therapeutic strategies for TBI-induced neurodegeneration

**DOI:** 10.3389/fneur.2025.1637243

**Published:** 2025-08-05

**Authors:** Anna O. Giarratana

**Affiliations:** Nuclear Medicine and Molecular Imaging Section, Department of Radiology, University of Wisconsin-Madison, Madison, WI, United States

**Keywords:** traumatic brain injury (TBI), PET/CT, PET/MRI, neurodegeneration, concussion, brain injury, neurotheranostics, precision medicine

## Abstract

This review examines the role of positron emission tomography (PET) imaging tracers in advancing our understanding of traumatic brain injury (TBI) induced neurodegeneration and the therapeutic targets they help to identify. It focuses on tracers used to evaluate post-TBI alterations in metabolism, amyloid, tau, neuroinflammation, and neurotransmitter systems. These molecular imaging tools provide critical insights into pathophysiological processes such as disrupted glucose metabolism, amyloid deposition, tau accumulation, chronic neuroinflammatory responses, and neurotransmitter dysregulation. The review also explores how these tracers, as imaging biomarkers, may guide future therapeutic strategies. Finally, it discusses the challenges and opportunities associated with integrating PET imaging into TBI diagnosis, longitudinal monitoring, and treatment planning.

## Graphical abstract

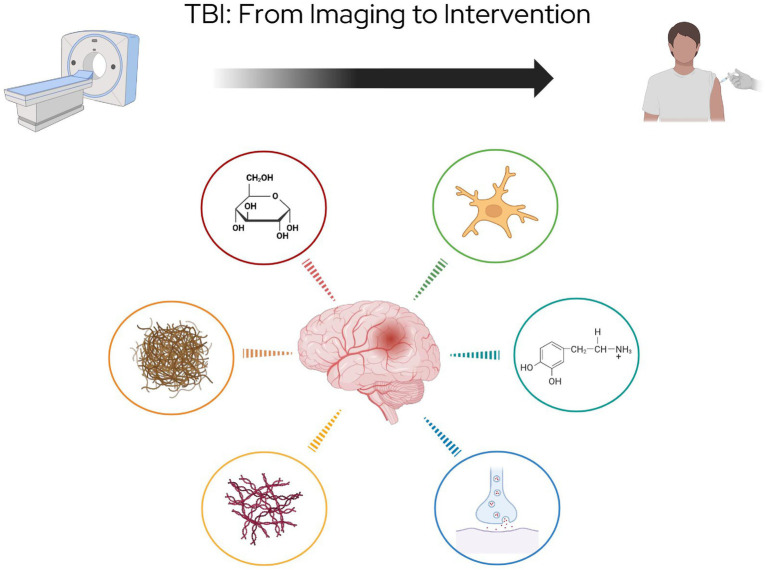

## Introduction

Traumatic Brain Injury (TBI) occurs when there is a disruption to normal brain function by an external mechanical force (1). This can be caused by a bump, a blow, or a jolt to the head as well as penetrating wounds (2, 3). TBI is a significant problem worldwide, with estimates that TBI affects up to 69 million people per each year (4, 5). TBIs are usually classified on a scale of severity, from mild to severe, and can have both short-term and long-term consequences (6). Despite the usual benign connotation of the word “mild,” mild TBIs, often colloquially referred to as concussions, can result in significant acute and chronic consequences, especially in the case of repeated mild injuries (7). In the cases of moderate and severe TBI, patients are usually so significantly affected that they require care in specialized neurological intensive care units (8, 9). New consensus guidelines were recently published that aim to more specifically classify TBI using a framework based on four pillars: clinical (full Glasgow Coma Scale with additional clinical components), biomarkers (blood-based markers such as GFAP, UCH-L1, and S100B), imaging (early CT findings), and modifiers (factors such as other medical conditions and psychosocial aspects). This framework addresses the limitations of the traditional severity scale by capturing injury complexity, but it remains in the early stages of implementation (10). For the remainder of this review, we will use the mild, moderate, and severe classification scale due to its prevalence in the literature (Graphical Abstract).

The effects of TBI are generally thought to occur in two phases: the acute effects occurring in the days and weeks immediately after the insult and the chronic sequelae often occurring years after the initial insult. In the acute phase of injury, the direct mechanical force from the injury results in both neuronal and axonal shearing, often termed the primary brain injury (11, 12). In this phase, a complex cascade of biochemical and cellular events is initiated. The long-term consequences of TBIs are often set into motion during the acute phase, with the activation of neurodegenerative processes leading to the secondary brain injury (13).

In the primary brain injury, there is often disruption of both neuronal and glial cells, axonal shearing, and vascular injury. This phase is primarily defined by the structural damage to the brain tissue, with apoptotic cell death, loss of synaptic density, cell membrane damage, blood brain barrier disruption, and an acute neuroinflammatory response as consequences (12, 14). The cellular and molecular cascade triggered by the primary brain injury leads to the chronic secondary brain injury and long term neurodegeneration (12). Apoptotic cell death and the loss of synaptic density disrupt neuronal networks, leading to cognitive and motor impairments that may persist beyond the initial injury (15). Blood–brain barrier and cell membrane disruption often results in excessive glutamate release, triggering excitotoxicity, mitochondrial dysfunction, and oxidative stress, all of which exacerbate neuronal loss and white matter damage (16–18). Additionally, axonal degeneration plays a crucial role in the accumulation of amyloid and tau pathology by disrupting intracellular transport, leading to abnormal protein aggregation (19, 20). The overactivation of the neuroimmune system further amplifies these processes, as sustained microglial activation drives chronic neuroinflammation, while astrocytic gliosis contributes to both early protective and late maladaptive responses (12). Together, these pathological mechanisms promote widespread neurodegeneration, progressive tauopathy, and amyloid deposition, increasing the risk of developing long term neurodegenerative disorders such as chronic traumatic encephalopathy (CTE).

It is vital to understand these underlying pathophysiological changes in the brain following TBI in order to develop targeted diagnostic and therapeutic strategies. While the exact mechanisms underlying TBI-induced neurodegeneration are not fully understood, there has been significant progress in recent years in advancing our understanding of the pathophysiology of TBI and developing more precise and advanced methods for evaluation and diagnosis. It is important to note, however, that one major challenge in this effort is the inherent heterogeneity of TBI, as differences in injury mechanisms, severity, and individual responses contribute to variability in both clinical presentation and the underlying metabolic changes.

Historically, research investigating TBI has relied heavily on biochemical assays and immunodetection methods, such as Western blot, ELISA, and immunohistochemistry, to identify pathophysiological pathways in both human and animal models (20–22). While these lab bench techniques remain essential, a promising new direction is the development of advanced imaging techniques, which provide greater spatial and functional insights into brain injury processes.

**Figure 1:**
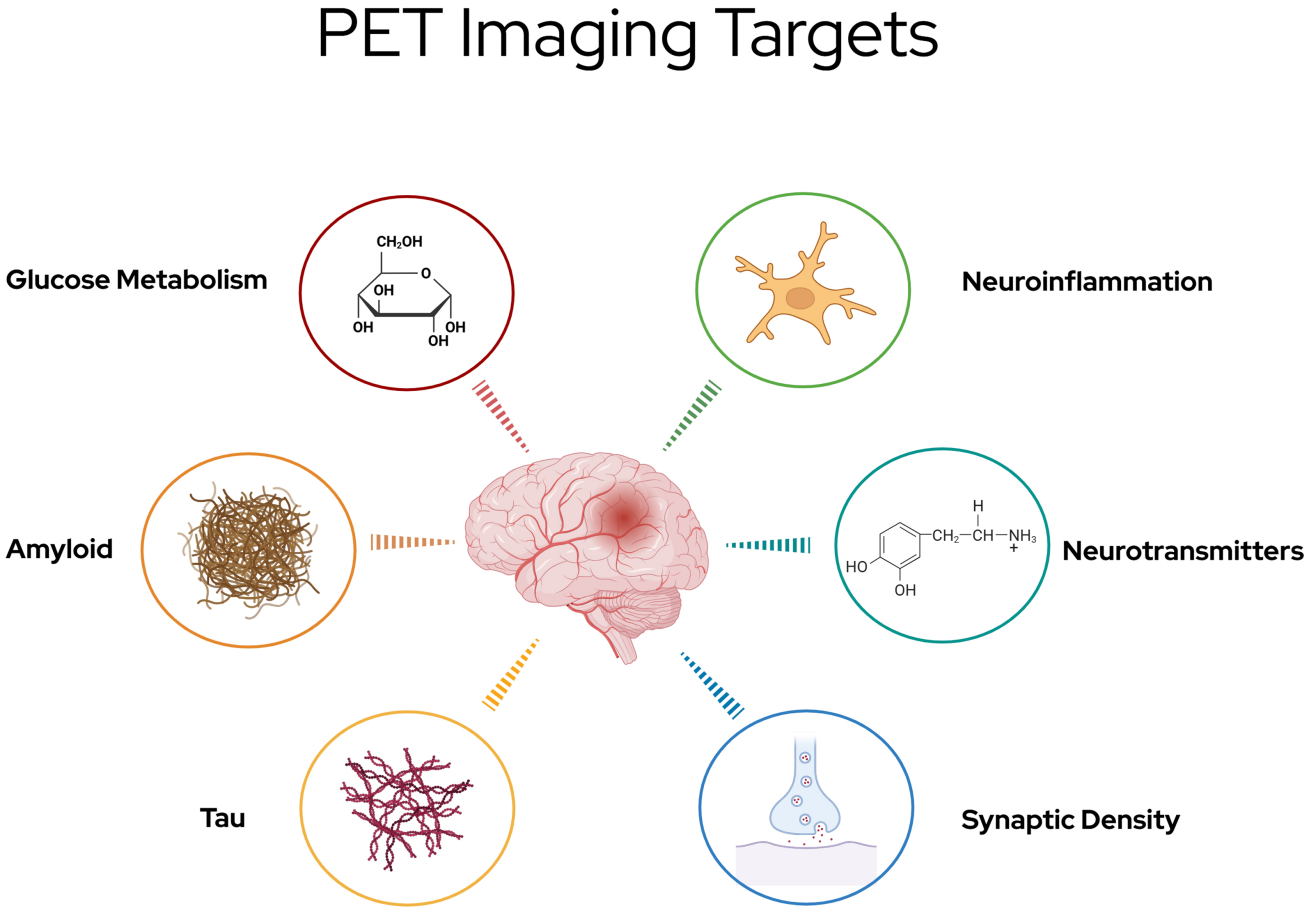
PET imaging targets in TBI, highlighting six major pathophysiological processes under investigation for evaluation with molecular imaging.

**Figure 2:**
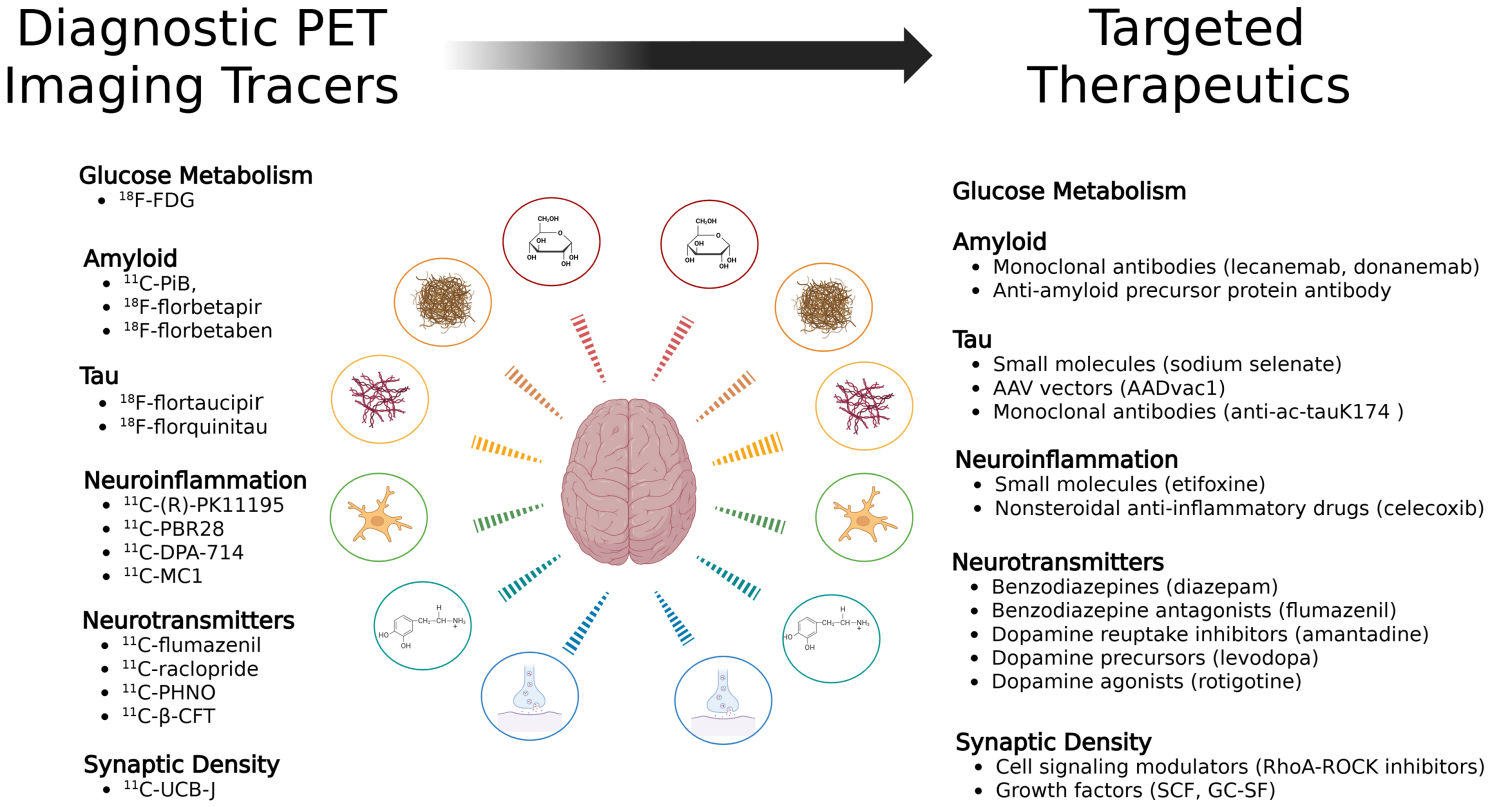
Diagnostic PET tracers and their potential corresponding targeted therapeutics mapped across TBI pathophysiological pathways.

This review will briefly discuss the role of anatomical imaging in TBI before shifting focus to physiological PET imaging. The majority of this review will explore the use of PET imaging in TBI research, with a specific focus on PET tracers, including those targeting metabolism, amyloid, tau, neuroinflammation, and neurotransmitters (Figure 1). These tracers provide critical insights into the progression of TBI-induced pathology and hold potential for guiding future therapeutic strategies. Next, the review will explore targeted treatments which show promise as a therapeutic pair to these tracers (Figure 2). Finally it will address methodological challenges, limitations, and opportunities for integrating PET into clinical practice for TBI.

## Search strategy

Searches of the PubMed database between February 2025 and May 2025 were conducted using the following keywords: “PET” OR “positron emission tomography” + “brain injury” OR “traumatic brain injury” OR “TBI” OR “concussion.” Searches of the clinical trials database at ClinicalTrials.gov were conducted using the following keywords: “PET” OR “positron emission tomography” + “brain injury” OR “traumatic brain injury” OR “TBI” OR “concussion.” Relevant papers were included in this narrative review.

## Neuroimaging in TBI

Traditional imaging techniques, such as computed tomography (CT) and magnetic resonance imaging (MRI), have played an important clinical role in the evaluation and diagnosis of TBI. CT scans are widely used in acute settings to detect major structural damage such as skull fractures and bleeding within the brain, however it has limited utility in evaluating more subtle and chronic changes in the brain parenchyma (23). MRI can provide a high-resolution image of the brain after injury, especially in regards to white matter damage and microhemorrhages, however standard MRI lacks the sensitivity to detect more subtle changes after injury (24).

Advances imaging techniques are largely in the research domain currently; however, effects are being made to develop clinically relevant and translatable advanced imaging techniques. Among these are advanced MRI imaging techniques, such as functional MRI (fMRI) and MRI Diffusion Tensor Imaging (DTI). fMRI detects changes in oxygen consumption to assess brain activity, aiding in the evaluation of cognitive function and disorders of consciousness, though it is not routinely used in acute TBI assessment. DTI maps white matter tracts to identify axonal injury and microstructural damage, offering potential prognostic value, though the findings remain highly variable and are not yet standardized. Other advanced MRI techniques utilizing perfusion, elastography, and spectroscopy are under evaluation but remain in the early stages of research (24).

One promising advanced imaging method is the use of positron emission tomography (PET), which, unlike CT and MRI which provide largely anatomical information, provides molecular-level insights into TBI-related brain changes. PET imaging uses radiotracers developed to specifically probe pathological processes such as glucose uptake, metabolic dysfunction, amyloid and tau deposition, neuroinflammation, and neurotransmitter dysfunction (25). PET imaging offers the advantage of quantitative tracking of physiological processes over time, making it a facile tool for monitoring progression of disease and monitoring of therapeutic response. The combination of physiological PET imaging with the anatomical CT or MRI imaging combines the advantages of these imaging methods, providing the dynamic pathophysiology of PET imaging with the superior spatial resolution of CT and MRI (26). In cases where CT and MRI fail to provide imaging findings that correlate with patient symptomatology, molecular imaging with specific PET tracers may offer promising alternatives (27).

## Diagnostic PET tracers in TBI

PET imaging utilizes pathophysiology to provide a molecular level view that can be leveraged for imaging purposes. In PET imaging, there are three main important components; a positron emitting radioisotope, the molecular component it is tagged to, and the radiation detectors used for imaging. The radioactive isotypes that are used for PET imaging are characterized by positron emission, which undergoes positron-electron pair annihilation, the result of which is two 511 kEV photons traveling approximately 180 degrees from each other. In PET imaging, the patient receives an intravenous injection of radiotracer. Depending on the molecular component, it is differentially taken up by tissues within the body. The radioactive isotope undergoes position emission, resulting in pair annihilation, and the two 511 kEV photons are detected by the detector ring encasing the patient. Using this information, the location of the radiotracer can be determined, and the combination of PET with CT or MRI imaging allows for precise anatomical localization of the radiotracer (28).

### ^18^F-fluorodeoxyglucose PET

The most frequently used PET tracer is ^18^F-fluorodeoxyglucose (^18^F-FDG). ^18^F-FDG, often referred to just as FDG, is an analog of glucose. As such, as a nuclear medicine imaging agent, it illustrates the *in vivo* physiological information about where glucose uptake is increased in the body. Traditionally, this has been valuable for oncology imaging, providing real time data on where glucose metabolism is so increased as to be suspicious for sites of hypermetabolic malignancy. Since its approval for oncology imaging in the late 1990s, the uses for FDG PET imaging have dramatically expanded. FDG PET imaging conjunction with CT and MRI has expanded into brain imaging, for indications such as dementia, epilepsy, and brain injury. While FDG PET imaging for dementia and epilepsy have entered the clinical use domain, FDG PET for brain injury still remains mostly in the research domain (29).

For brain injury, FDG PET has been used in order to probe metabolic changes in the different stages by evaluating regional glucose metabolism, a proxy for neuronal viability. As discussed, TBI is usually thought to occur in two stages, the acute or primary brain injury, and the chronic or secondary brain injury. In general, FDG PET has demonstrated patterns of both hypometabolism and hypermetabolism, depending on the time course between the injury and imaging, the original severity of the injury, and the regions of the brain that were impacted by the injury. Clinical and preclinical studies have used various methodologies to investigate mild, moderate, and severe brain injuries, at the acute, subacute, and chronic time points after injury. Despite differences in methodology, patterns have emerged that show that generally, in the acute to sub-acute stages, there can be either hypermetabolism signifying transient excitotoxic activity, or hypometabolism, signifying functional impairment of brain parenchyma. In the sub-acute to chronic stages, there is nearly always hypometabolism, indicating long term structural and functional damage to brain tissue (29). With more research, the ability of FDG PET to detect metabolic changes after injury may be a valuable tool for characterizing injury severity, predicting outcomes, and informing therapeutic strategies.

Both clinical and preclinical research have investigated the use of FDG-PET in TBI, with clinical studies examining imaging in relation to patient outcomes and preclinical research exploring mechanistic insights that are more difficult to determine in human subjects. Clinical studies often categorize patients based on TBI severity, typically focusing on either mild or moderate to severe TBI. Among studies that recruited patients with TBI of varying severity (mild, moderate, and severe), injury mechanisms included motor vehicle accidents, falls, and blast-related impacts. Few studies enrolled patients in the acute to subacute phase, while the majority focused on chronic-phase TBI. Both men and women were included, though men were overrepresented in certain populations, particularly in military cohorts where blast-related TBIs were investigated.

Early FDG PET studies in mild–moderate TBI demonstrated regional metabolic alterations, primarily hypometabolism in the frontal and temporal lobes, often correlating with cognitive dysfunction. The first of these found regional hypometabolism in the posterior temporal cortex, the and frontal cortex, and the caudate nucleus, with some evidence of increased metabolism in anterior cortical regions (30). Later studies provided further evidence for hypometabolism in the frontal and temporal lobes in chronic mild TBI cases (31, 32). However, the sample sizes in these earlier studies were very small, ranging from *n* = 3 to *n* = 20.

More recent FDG PET clinical studies have focused on the military population, and in particular blast related TBIs which have increased in frequently due to developments in modern warfare. These studies have found that in mild to moderate blast TBIs, that there are disruptions in metabolism not just in the cortex as previously seen, but widespread. These studies have found hypometabolism in the cerebellum and medial temporal lobe in veterans exposed to repetitive blasts (33), hypometabolism in the parietal, somatosensory, and visual cortices (34), as well as worse hypometabolism in the right superior parietal region in blast injuries relative to blunt force injuries (35).

In addition to these studies of mild to moderate brain injuries, there have been a number of clinical studies investigating FDG PET in moderate and severe brain injuries as well. One early study investigated the full spectrum from mild to severe brain injuries and found that across the spectrum of injury, that patients had 88% regional reductions in cerebral glucose metabolism. They found that in the more severe cases, that there were more prevalent global cortical reductions (36). Another early study found that metabolic activity in the hemispheric cortical gray matter was elevated immediately following injury, but that by 1 month following injury, most of these changes had resolved back to baseline across all groups of injury level severity. Another early study found that metabolic activity in the hemispheric cortical gray matter was elevated immediately after injury until around 5 days post-injury, followed by a period of reduced cortical metabolism from 5 to 28 days post-injury. However, by 1 month post injury, most of these changes had resolved back to baseline across all injury severity levels (37). Another study found that, interestingly, when patients were stratified into mild and severe TBI groups based on MRI findings, those with severe TBIs and visible lesions on MRI demonstrated significantly decreased metabolism in the orbital gyrus, cingulate gyrus, and medial thalamus, but increased metabolism in the parietal and occipital convexity compared to controls. In contrast, those with mild TBIs without MRI findings exhibited no significant metabolic changes (38). However, the time course from the time of injury has been found to be important. One recent study looking at FDG-PET in veterans with a remote history of mild to severe TBI found that no significant differences in FDG uptake were observed between veterans with service-related TBI and controls after adjusting for covariates (39).

Despite the fact that TBI is a heterogeneous pathology, these studies have provided evidence for hypometabolism in areas of the brain such as the frontal, temporal, and cingulate regions after mild brain injury. On the other hand, moderate and severe TBI studies have shown hypermetabolism acutely after injury early in the cortex, the parietal, and occipital regions. This is followed by widespread hypometabolism similar to what is seen in mild TBI (37, 40–42). While these general patterns have been seen in the literature, it is important to stress that as heterogenous as TBI is as a disease, so is the FDG uptake patterns seen after injury.

It is important to note that methodological inconsistencies, variations in injury mechanisms, and differences in normalization techniques complicate cross-study comparisons. One important point of contention is whether or not there is clear evidence for hypermetabolism at any point in the disease course. There is limited evidence suggesting that hypermetabolism may occur immediately after injury in patients; however, further studies focusing on the acute phase are needed (37). Enrollment challenges often make such studies difficult to conduct.

Review of the clinical trial database on ClinicalTrials.gov shows 6 studies currently listed evaluating “FDG + PET” for “traumatic brain injury.” Two of these are completed, one is recruiting, one is enrolling by invitation, one was terminated, and one has an unknown status. Focusing on the completed studies, the goal of the first (NCT02424656) was to identify reliable biomarkers of consciousness recovery following TBI by examining longitudinal changes in whole-brain connectivity using multimodal neuroimaging (43). However, results published from the study so far have not utilized analysis of the FDG PET data (44, 45). The goal of the second completed study (NCT04730167) was to evaluate retired professional motorsport drivers, a population with high past exposure to brain trauma, for the long-term effects of TBI; however, no results have been published referencing this trial to date. Given the limited number of registered clinical trials, incomplete published results, and heterogeneous clinical data, along with the high cost and complexity of clinical research, preclinical studies offer a valuable and cost-effective opportunity to refine our understanding of the role that FDG PET can play in patient evaluation after TBI.

Preclinical studies are essential for addressing questions that are challenging to investigate in human populations. They provide a more accessible platform for studying the acute phase of injury and exploring the underlying pathophysiology in greater detail. Experimental models of TBI include controlled cortical impact (CCI) (46), lateral fluid percussion (LFP) (47), as well as shockwave and blast models of TBI (48). However, preclinical studies investigating FDG PET changes after TBI are still relatively rare, mainly due to technical constraints like limited access to small animal PET scanners and the difficulty of detecting subtle metabolic shifts after injury.

The preclinical studies that have been done span mild to severe injury severities. One study using the LFP method to induce mild TBI found around a 15% drop in FDG PET uptake at 24 h post-mild LFP, with recovery by 9–15 days, correlating with astrocytosis and axonal injury. In this study, they did not note any periods of hypermetabolism after injury (49). Other studies using moderate or severe injury models have also shown more consistent evidence of acute and sustained cerebral hypometabolism. In moderate injury models, one group found that FDG PET uptake typically returned to normal within 10 to 14 days after TBI induced via the Freeney weight drop model (50). On the other hand, another study using a severe LFP injury model found that there were prolonged reductions in FDG uptake, with changes observed in the ipsilateral cortex, hippocampus, and amygdalae at 1 week, 1 month, 3 months, and 6 months post-injury (51). While most of these studies did not include an acute early-phase time point to assess potential transient hypermetabolism, one study using a shockwave model of TBI evaluated FDG PET uptake at 3 h post-injury. The data showed ipsilateral hypermetabolism at 3 h, which resolved to non-significant levels by 3 days (52). This finding underscores the need for further longitudinal studies that incorporate acute early-phase time points to better evaluate potential hypermetabolism after injury. Overall, preclinical animal models have shown that mild, moderate, and severe TBIs all result in hypometabolism, with faster recovery to baseline in mild TBI compared to moderate and severe cases, which can exhibit prolonged hypometabolism lasting at least 6 months post-injury. While there is some evidence for a transient hypermetabolic phase acutely after injury, this phenomenon is not well characterized and requires further study to determine its significance and underlying mechanisms.

These preclinical findings both reinforce and extend clinical observations after TBI. They provide additional evidence for hypometabolism in chronic TBI and clarify the time course and regional specificity of metabolic changes across injury severities. Notably, the preclinical studies enhance our understanding of the temporal dynamics of FDG PET signal, showing evidence of transient hypermetabolism in the early post-TBI phase. This helps explain inconsistencies in clinical data and underscores the importance of early imaging windows that are challenging to capture in human studies.

In summary, FDG PET imaging in both clinical and preclinical studies has provided valuable insight into the metabolic consequences of TBI across injury severities and time points. While hypometabolism is the most consistently observed pattern, particularly in the chronic phase, there is some evidence that suggests a possible transient hypermetabolic phase acutely after injury. Building on the findings from early FDG PET studies, researchers have begun using newer PET tracers, such as those targeting amyloid and tau, to investigate neurodegenerative changes after injury in a more nuanced and targeted manner. However, there are limitations to the use of FDG PET imaging in TBI. Most notably, the lack of specificity for pathology. FDG uptake reflects general metabolic activity and cannot distinguish between neurons, glia, or inflammatory processes, which may limit its interpretive value in heterogeneous TBI presentations.

### Amyloid PET

A relatively new class of PET radiotracers targets amyloid-beta (A*β*) peptides, enabling *in vivo* detection and quantification of amyloid accumulation in the brain (53). Under normal conditions, amyloid precursor protein (APP) undergoes proteolytic cleavage through two main pathways: the non-amyloidogenic pathway, initiated by *α*-secretase, and the amyloidogenic pathway, initiated by β-secretase. Subsequent cleavage by *γ*-secretase produces either non-toxic P3 peptides or Aβ peptides, including Aβ40 and Aβ42, which are associated with amyloid pathology (54). Aβ peptides self-assemble into various aggregate forms, including soluble oligomers, protofibrils, and insoluble amyloid fibrils (55, 56). While oligomers can diffuse throughout the brain, fibrils accumulate into plaques. Both amyloid fibrils and plaques have been implicated in synaptic dysfunction, microglial activation, and progressive neurodegeneration (57).

Amyloid PET tracers have been developed with high sensitivity and specificity to fibrillar Aβ aggregate (58). The first successful imaging of amyloid plaques was achieved in 2004 using the radiotracer ^11^C-labeled Pittsburgh compound B (PiB) (59). Since then, a number of different ^18^F labeled tracers, such as florbetapir (also known by the name Amyvid) (60), florbetaben (also known by the name Neuraceq) (61), and flutemetamol (also known by the name Vizamyl) (62) have been developed to facilitate widespread and practical imaging of amyloid pathology, owing to their longer half-life and suitability for off-site production and distribution (63). In addition to dedicated amyloid agents, UCLA has developed a PET imaging tracer called 2-(1-[6-[2-^18^F-fluoroethylmethylamino]-2-naphthyl]ethylidene)malononitrile, known as ^18^F-FDDNP, which binds to both amyloid and tau in the brain (64, 65).

Although primarily studied in Alzheimer’s disease, amyloid accumulation has also been reported in other neuropathological contexts, such as in TBI, where the secondary amyloidogenic cascades may be initiated (66). Amyloid PET provides a noninvasive tool to study Aβ deposition and its broader implications in neurodegenerative and injury-related brain disorders. In TBI, amyloid PET has been used to investigate the potential accumulation of fibrillar Aβ as a marker of secondary neurodegenerative processes (67). Although amyloid deposition is not typically present in the acute phase, it may emerge in the subacute to chronic stages, particularly in moderate to severe injuries or those involving repetitive trauma (68).

Both clinical and preclinical studies have investigated amyloid PET imaging in relation to TBI. While many studies suggest that amyloid deposition increases following TBI, the findings have been inconsistent regarding the magnitude of the effect and the specific brain regions involved (69). The regions that have had the strongest evidence for involvement have been the frontal lobes, cingulate cortices, the precuneus region, and the cerebellum. Early studies using PiB found that patients with TBI had higher binding of PIB throughout the brain, but specifically in the cortical grey matter and striatum (70). Another study using PiB evaluated individuals with a history of TBI, to those with a diagnosis of AD, as well as to healthy controls. They found that amyloid deposition was increased in the posterior cingulate cortex and cerebellum compared to controls. They saw that the pattern of amyloid deposition differed in people with a history of TBI compared to the people with AD, with lower neocortical but higher cerebellar binding, suggesting that TBI may promote distinct amyloid pathology (71). Another study, using florbetapir, investigated amyloid distribution in patients with TBI, PTSD, and the combination of both. Interestingly, TBI and PTSD were associated with different patterns of amyloid deposition. TBI patients demonstrated elevated amyloid primarily in the precuneus and cerebellum, while PTSD patients showed more widespread amyloid accumulation across cortical regions (72). Although, it must be noted, that subsequent reanalysis of the same dataset by a different group were unable to replicate these regional findings, raising questions about the reliability of the original voxelwise results (73). Although the authors themselves re-analyzed the data and did seem to find similar results to their original data, they remark on the sensitivity of their findings to methodological choices and acknowledge that the small effect sizes approach the detection limits of current amyloid PET tracers (74).

There is also increasing evidence that the regions of the brain that are affected by amyloid deposition will vary by injury type. For example, diffuse axonal injury is a very specific type of injury. In this case, studies have found that there is increased amyloid deposition in the occipital and temporal cortices compared to controls, along with occipital atrophy (75).

In addition to injury type, the relationship between TBI and amyloid burden appears to be more pronounced in individuals with either repetitive or more severe injuries or who have concomitant cognitive impairment, highlighting the potential importance of injury severity and clinical phenotype in modulating amyloid pathology post TBI. One group found that in a study of 329 participants without dementia, that TBI was associated with an increase in amyloid deposition in the brain, measured by florbetapir. Interestingly, they also found that there was a dose response relationship between the two, where patients with more than one TBI had elevated amyloid deposition in the prefrontal cortex and superior frontal cortex relative to patients with a history of one head injury (76). Another study conducted a prospective evaluation of a cohort of military instructors exposed to repeated sub-concussive blast injuries and age and sex matched healthy controls without injury exposure or history. They found that in the cohort that had exposure to repeated sub-concussive blast TBIs, that within 5 months, there was amyloid-*β* accumulation in the inferomedial frontal lobe, precuneus, anterior cingulum, and superior parietal lobule as evaluated by florbetapir (77). An additional study evaluated patients with a TBI history within the previous 6 years. They included mild TBI patients with and without cognitive impairment, as well as healthy controls. They found that there was increased PET amyloid deposition and higher APOE ε4 frequency in the mild TBI group with cognitive impairment, suggesting that amyloid accumulation may contribute to post-TBI cognitive decline. These findings support a possible link between TBI and dementia risk, potentially mediated by APOE ε4. However, because amyloid was elevated only in those with cognitive decline, the findings may reflect dementia related pathology, rather than being a direct effect of TBI alone (78). Another group studied 241 individuals with a remote history of TBI, and found that in this group, there was an earlier onset of cognitive impairment by three to 4 years relative to those without a TBI history. Evaluation with florbetapir showed that among those who were amyloid positive, that those participants with a TBI history had greater amyloid deposition in the cortex, providing further evidence for the idea that suggesting that remote TBI may accelerate amyloid accumulation and neurodegeneration along the Alzheimer’s disease continuum (79).

On the other hand, there have been several large studies that have shown no correlation between TBI and amyloid deposition as measured by PET. One study evaluated a cohort of 237 men, including former professional and college American football players and unexposed controls. They found that, using florbetapir, there were no significant differences in amyloid deposition between the TBI group and the controls, even in the those with repeated TBIs (80). In another study, they found that in a study of 70 Vietnam War veterans, both with and without TBI history, that there were no significant differences in amyloid deposition, after adjusting for age and APOE ε4 status (39). Another group found that in 134 older adults, that those with a remote history of mild TBI did not have an association with greater amyloid deposition (81). Given the known differences between mild and moderate to severe TBI, these findings are not entirely unexpected. However, they do provide support for the idea that the combination of moderate to severe TBI, genetic risk factors, and underlying cognitive vulnerabilities may accelerate amyloid pathology. It is also important to consider differences in binding properties among amyloid tracers. While the ^18^F-labeled tracers offer advantages over the original ^11^C-based PiB tracer, particularly in terms of half-life and broader clinical applicability, there may be limitations in their sensitivity for detecting subtle amyloid differences, especially outside of typical Alzheimer’s disease pathology (82). In addition, as the majority of TBI studies are retrospective rather than prospective in design, they are inherently limited by potential recall bias, particularly when exposure events occurred more than five decades earlier.

However, there are a number of clinical trials currently listed that are using amyloid PET in their evaluation in some way. Review of the clinical trial database on ClinicalTrials.gov shows 16 studies currently listed evaluating “amyloid + PET” for “traumatic brain injury.” Of the 16 listed studies, 7 have been completed, 1 is recruiting, 1 is enrolling by invitation, 1 is not yet recruiting, 1 was terminated, 2 were withdrawn, and the status of 3 are unknown. Many of these studies utilized amyloid, sometimes in conjunction with tau, which will be further discussed in the next section, to evaluate for outcomes after TBI. Focusing on the completed studies, two of these have been posted with results. The first of these (NCT02003183) aimed to evaluate if PET imaging with the combined amyloid and tau PET tracer, ^18^F-FDDNP, could be used to study patterns of amyloid and tau pathology in individuals with a medical history significant for repeated TBIs and suspicion for CTE. They wanted to determine if individuals with suspected CTE had unique patterns of PET activity from both healthy controls and those with AD, which would provide support for this tracer as an imaging biomarker for *in vivo* analysis of CTE, a diagnosis currently made at autopsy. The study group has published multiple papers from this trial, one which showed that ^18^F-FDDNP PET imaging in the suspected CTE group was more extensive in the subcortical regions and the amygdala compared to controls, however they point out that this study was limited by their small sample size and lack of autopsy confirmation (83). This group also published results showing that ^18^F-FDDNP tracer uptake reflects neuropathology in brain regions most affected by repetitive head trauma (84); that ^18^F-FDDNP detects *in vivo* tau and amyloid brain signals in military personnel with mild TBI similar to those seen in retired football players but distinct from AD (85); and that ^18^F-FDDNP PET findings in a retired player correlated with postmortem tau deposition, supporting its diagnostic potential (86). The second completed study with results (NCT02191267) used the amyloid tracer, florbetapir, as a control for their study which focused on the tau tracer, flortaucipir. Briefly, they aimed to study tau accumulation in a group of retired NFL players with suspected CTE relative to athletes without brain trauma as well as to individuals with AD. In regards to the amyloid results, they expected to see amyloid burden in the AD patients but not the suspected CTE group or the healthy athlete control group. While the authors have published other reports on the tau tracer (87, 88), no studies appear to have been published on the trial results, which seem to indicate that the AD did indeed have more amyloid and tau PET activity than either the CTE or healthy athlete control group, although the tau specific hypothesis may not have been borne out given the preliminary data available.

Another trial (NCT02266563) aimed to investigate whether people with a past medical history significant for TBI have similar accumulations of amyloid, using florbetapir, and tau to those with AD. The results of this study have not yet been posted, although the study sponsor has since published research focusing on tau accumulation after TBI (89). Other studies that have been completed but without posted results have planned to study amyloid PET as part of a larger cohort of biomarkers (NCT04928534), as a means of correlating disease severity in a tau PET tracer study (NCT02103894), as an outcome measure in a CTE trial (NCT02798185), and as part of an evaluation of whether TBI related axonal injury results in increased amyloid accumulation (NCT01687153).

While several clinical trials have incorporated amyloid PET to evaluate outcomes after TBI, the data are far from conclusive. Given the use of the tracer ^18^F-FDDNP which has both amyloid and tau uptake, it is difficult to tease apart the role of each of these agents based on the data presented. Other studies have presented evidence that amyloid is particularly elevated in AD but less so after TBI, even in cases of suspected CTE, and highlight the role for tau PET in these cases.

While preclinical PET studies in TBI models using FDG was relatively rare, amyloid PET studies are even less common. In one particularly thorough preclinical study, they evaluated the long-term effects of TBI, using both the CCI and LFP models of injury, on amyloid accumulation in a mouse model of Alzheimer’s disease. They used a radiotracer that was specific for Aβ protofibrils, termed ^124^I-RmAb158-scFv8D3. They found that while there were no differences between the groups at the 12 week time point after injury, that by the 24 week time point, that injured mice had significantly more amyloid deposition in the frontal cortex relative to uninjured controls, and that this correlated with histology markers showing more reactive gliosis at that time point as well (90). These findings suggest that the neurodegenerative processes initiated by traumatic injury may contribute to the delayed accumulation of amyloid pathology over time and highlight the importance of preclinical trials in evaluating these processes in a prospective manner.

The preclinical data supports the clinical findings of delayed or chronic amyloid accumulation after TBI and highlights how injury severity and chronicity influence amyloid deposition. By linking amyloid PET signal with histology, animal models clarify mechanisms that may be masked by variability in clinical populations, including age, APOE ε4 status, and comorbid pathologies.

In summary, amyloid PET imaging enables *in vivo* detection of Aβ peptides and has been explored in both clinical and preclinical studies of TBI to assess neurodegenerative after TBI. While some studies have reported increased amyloid deposition following moderate to severe or repetitive TBI, particularly in regions like the cerebellum, precuneus, and frontal cortex, findings remain inconsistent. Preclinical models suggest that amyloid accumulation may emerge after injury in a delayed time course, underscoring the need for longitudinal and mechanistically grounded studies. Clinical trials using amyloid PET, often alongside tau PET, suggest that amyloid accumulation is more prominent in Alzheimer’s disease than in CTE or TBI, reinforcing the value of tau imaging in distinguishing these conditions. However, amyloid PET has important limitations in TBI, including inconsistent findings across studies and the potential for elevated amyloid signal to reflect preclinical Alzheimer’s pathology rather than a TBI-specific process, particularly in older individuals or APOE ε4 carriers.

### Tau PET

Another newer class of PET radiotracer targets aggregated tau protein. Tau protein is a microtubule-associated protein that is synthesized throughout the nervous system that, under normal conditions, plays an important role in stabilizing microtubules and supporting the cytoskeleton, enabling the essential intracellular transport of nervous system components such as secretory vesicles and neurotransmitters (91). Under these normal conditions, phosphorylation plays an important role in activating the tau protein. However, in pathological states, tau can become hyperphosphorylated which can result in the dissociation and aggregation of tau into neurofibrillary tangles (92). There have been a number of tau tracers developed which have been developed to selectively bind to the tau neurofibrillary tangles. Tau tracers can be characterized into first and second generation. The first generation includes tracers such as ^18^F-AV-1451 (flortaucipir), ^11^C-PBB3, and the ^18^F-THK family, which were effective in binding to tau, however exhibited significant off target binding to regions such as the choroid plexus, the basal ganglia, and the meninges (93). The second generation was developed to increase tau specific binding and minimize off target binding, and includes tracers such as ^18^F-MK-6240 (florquinitau), ^18^F-RO948, ^18^F-PI2620, ^18^F-GTP1, and 18F-JNJ-64326067 (91, 94, 95). While initially developed for classic neurodegenerative disorders such as AD, researchers have started to utilize tau PET imaging to probe the neurodegenerative sequela that follow TBI.

As previously discussed, after the acute phase of TBI which involves metabolic and structural disruptions, the chronic phase of TBI is set into motion by a cascade of neurodegenerative processes. One aspect of this process involves the development tau hyperphosphorylation resulting in neurofibrillary tangles. This is particularly true in the case of repeated TBIs and is thought to be a mediating factor in the development of CTE (96). Tau PET offers us a novel noninvasive way of assessing the *in vivo* development of tau neurofibrillary tangles, previously assessed similar to amyloid pathology, only at autopsy. By studying the accumulation of tau PET longitudinally after TBI, researchers hope to better understand the role that tauopathy has in the clinical symptomology post TBI, the spatial and temporal dynamics of tau pathology, and to identify interventions that may halt this progression.

A number of clinical studies have investigated tau PET imaging in relation to TBI. The clinical data from studying tau PET after TBI have been more consistent than those investigating amyloid. Studies investigating tau PET in former NFL players with concern for CTE found that in this group, relative to controls, there was higher flortaucipir PET signal in regions known to be involved in CTE, including the superior frontal, medial temporal, and left parietal cortices. They also found that the tau PET signal had a correlation with years of football play, but not symptom severity, perhaps indicating the role that unrealized sub-concussive repetitive injuries play in the development of tauopathy. In addition, in this study they also investigated amyloid PET using florbetapir and found that there was no elevation in amyloid PET imaging in this population, providing evidence that TBI, and in particular CTE, may be driven by tauopathy rather than amyloid pathology, which appears more specific to AD (97).

This group published the larger DIAGNOSE CTE Research Project results recently, with recently findings from a study using flortaucipir tau PET in 218 males, consisting of retired professional American football players, former college American football players, and 56 healthy controls. They found that tau PET uptake was significantly increased in the exposed American football players relative to controls, especially in the superior frontal, medial temporal, and parietal cortices. However, they did not see a correlation between the estimated cumulative head impact exposure in the main analysis, and only found a positive subgroup analysis for a correlation in the superior frontal region in those over 60 years of age (98). These findings support the utility of tau PET for *in vivo* detection of tau pathology; however, the lack of consistent correlation with estimated cumulative head impact exposure underscores the need for further studies to evaluate its utility as a marker of progressive disease.

A more recent study from a group with overlapping authors to the prior two studies found that the second-generation tau PET tracer ^18^F-MK-6240, also known as florquinitau, binds with high affinity to tau pathology in postmortem brain tissue from individuals with confirmed CTE. They found that *in vivo* there was variable uptake among their group of retired professional American football players with concern for CTE. Of the 29 participants, 13 showed cortical signal, while 16 had no cortical signal. Of those that showed cortical signal, 7 had medial temporal lobe uptake, 2 had frontal uptake, and 4 had both medial temporal lobe and frontal uptake. They found that there was a correlation in this group between increased tau PET signal and memory and language performance. Of note, this group was also found to have negative amyloid PET pathology (99). This study provides further evidence supporting tau PET as a potential *in vivo* biomarker for tauopathy following TBI and suggests that amyloid PET may have limited utility in this population.

Another group investigated tau PET in a small study with 11 male patients who were diagnosed with CTE, who sustained from repetitive head injuries while playing sports. In the amyloid negative subgroup of 9 individuals, they found that there was mildly elevated flortaucipir tau PET signal with a frontotemporal distribution with activity seen in the medial temporal lobes bilaterally. In the amyloid positive subgroup of 2 individuals, they saw severe brain atrophy on MRI and increased tau PET binding. These findings provide further evidence for tau PET as a possible biomarker for CTE, however the authors caution that it may not be sensitive to the disease in early states (100).

An additional group investigated the first generation tau PET tracer ^18^F-THK5317 in conjunction with a PET tracer for neuroinflammation and microglial activation, ^11^C-PK11195, in a mixed sex, young adult population. This study was unique in including both female and male participants, as well as in the younger average age of the cohort, approximately 26–27 years. They found that in their sports related concussion and TBI groups, that there was increased tau PET signal in the thalami, temporal white matter and the midbrain and increased neuroinflammation PET signal in the temporal white matter, hippocampus and corpus callosum (101). This study provides further evidence for both tauopathy and neuroinflammatory processes following TBI and demonstrates that these findings occur across genders and in young adulthood after TBI exposure.

As detailed here, tau PET imaging studies in TBI have shown more consistent findings than amyloid studies, suggesting that tau deposition plays an important role in the neurodegenerative processes that occur post-TBI and may play an important role in the long-term consequences that develop, such as CTE. These studies have highlighted tau deposition in a frontotemporal pattern, with increased tau PET tracer uptake seen in regions such as the medial temporal lobes, superior frontal cortex, parietal cortex, temporal white matter, midbrain, thalami, and corpus callosum. These spatial patterns overlap with known regions affected in CTE and provide preliminary evidence for tau PET as a method to track *in vivo* tau accumulation after TBI.

Most of the reported literature have used the first-generation tau tracer 18F-AV-1451 (flortaucipir), although one used the first-generation tracer of ^18^F-THK5317, and one used the newer second generation tracer ^18^F-MK-6240 (florquinitau). Given the differences in specificity and off target binding that has been studied between the different tracers, it will be important to conduct further research assessing both the sensitivity and specificity of each tau tracer in the context of TBI related tauopathy, particularly across different stages of disease and across diverse populations.

Many of the clinical trials listed that evaluate tau PET also evaluate amyloid PET and were therefore discussed in the prior section. Review of the clinical trial database on ClinicalTrials.gov shows 21 studies currently listed evaluating “tau + PET” for “traumatic brain injury.” Of the 21 listed studies, 10 have been completed, 4 of which have results, 1 is active, not recruiting, 2 are enrolling by invitation, 2 are recruiting, 1 is not yet recruiting, 1 has been terminated, 3 have been withdrawn, and 1 has a status unknown. Focusing on the completed studies, 7 of these overlap with the previously discussed clinical trials which also investigated amyloid PET (NCT04928534, NCT02798185, NCT02266563, NCT02191267, NCT02103894, NCT02003183, and NCT01687153). Focusing on the three remaining studies, two of these have posted results. The first study (NCT02278367) was run by Avid Radiopharmaceuticals and aimed to expand the flortaucipir PET safety and tau specific binding dataset by evaluating 179 individuals with and without cognitive impairment. Although there are no published papers linked to this entry, the posted results show evidence for a higher mean flortaucipir SUVr in cognitively impaired individuals relative to the cognitively normal controls, providing evidence for their intended indication. The second study (NCT02079766) was also run by Avid Radiopharmaceuticals and aimed again to evaluate the safety and imaging characteristics of flortaucipir PET, this time in a study 41 individuals with and without a history of TBI. In the results section, they listed an increase in mild flortaucipir PET uptake in the TBI group relative to controls. The publications that resulted from this study were previously discussed as the first clinical results in this section (97). The third study (NCT05183087) has no reported results as of yet, but hopes to identify biomarkers from TBI after low level blast injury, and includes tau PET using ^18^F-MK6240 as an outcome measure.

Again, preclinical studies evaluating tau PET in animal models following TBI have been limited. One study published an interesting report combining data from rats with those from humans. Using flortaucipir tau PET, they used a blast model of TBI in rats as well as human veterans were who exposed to blast related TBIs. They found that in their rat model, those with blast TBI exposure had increased levels of hyperphosphorylated tau in the anterior cortex and hippocampus, with perivascular tau accumulation observed in astrocytic processes. In the humans, they found that half of the blast exposed veterans had focal tau PET uptake in the frontal, parietal, and occipital cortices. They also noted a positive correlation between tracer uptake and neuropsychiatric symptoms (89). This study provided unique evidence, from their combined rodent and human data, supporting the role of tau PET as an *in vivo* biomarker for CTE-related tauopathy.

The combined preclinical and clinical findings suggest that TBI may initiate a cascade of events, which leads to chronic tauopathy, particularly in cases of repetitive TBI. Animal models offer unique mechanistic insights, providing histological evidence for perivascular tau accumulation and astrocytic involvement. These preclinical findings align with postmortem human CTE pathology and strengthen the evidence supporting tau PET as a translational biomarker.

In summary, tau PET imaging demonstrates a stronger and more consistent association with TBI related pathology than amyloid PET. Elevated tau signal has been observed in regions commonly affected in CTE, including the superior frontal, parietal, medial temporal lobes, and precuneus cortices. These findings support tau PET as a promising *in vivo* biomarker for post TBI neurodegeneration. However, there are limitations to the use of tau PET in TBI. Variability in tracer uptake, off-target binding, small sample sizes, and clinical heterogeneity all limit the interpretability of current studies. In the future, larger and longitudinal studies are needed to clarify the temporal dynamics of tau accumulation and the potential role of tau PET in diagnosis, prognosis, and therapeutic monitoring in TBI populations.

### Neuroinflammation PET

Another up-and-coming class of PET radiotracers has been developed to target neuroinflammation. Given the large role that the neuroimmune system plays after TBI, it follows that this would be an area of interest for PET imaging tracers to target. As discussed previously, after TBI, acute inflammation is part of the normal, physiological response aimed at repairing the brain. However, often, especially after severe or repetitive brain injuries, the neuroimmune response can become chronically activated and pathologic, resulting in secondary injuries. These neuroimmune responses are often mediated by activated microglial driving chronic neuroinflammatory responses and astrocytes contributing to astrocytic scarring and gliosis. From this understanding of the neuroimmune sequelae of TBI, researchers have been interested in developing PET agents to target the neuroimmune system.

The most common PET radiotracer target for neuroinflammation is the translocator protein (TSPO), which is upregulated in microglia and, to a lesser extent, astrocytes (102). TSPO is an 18 kDa outer mitochondrial membrane protein, that under normal circumstances, has low expression in the nervous system. However, in situations where microglia are activated, such as after injury or during neuroinflammation, there is a resultant increase in TSPO levels (103, 104). There have been a number of different tracers developed to target TSPO. The first-generation tracer ^11^C-(R)-PK11195 has been most extensively used, however has been hampered by its low signal to noise, given that TSPO is not only expressed in glial cells like microglia and astrocytes, but also in endothelial cells and peripheral immune cells. The second generation of tracers were developed to increase this signal to noise. These second-generation tracers include agents such as ^11^C-PBR28, ^11^C-DPA-713, and ^11^C-ER176. While the second-generation tracers have improved signal to noise, they have an additional issue of impaired binding in individuals with the rs6971 polymorphism (105). Studies in patients using these radiotracers therefore require genotyping for this genetic polymorphism for complete evaluation. Of the second-generation radiotracers, ^11^C-ER176 has emerged as a leading candidate due to its relative higher signal to noise and decreased sensitivity to the rs6971 polymorphism, although to date there are no published papers exploring this tracer in TBI (102, 106).

In addition to TSPO, there has been interest in developing other tracers targeting neuroinflammation in the brain. These include cyclooxygenase-1 (COX-1) and COX-2, colony stimulating factor 1 receptor (CSF1-R), and the P2X purinergic receptor 7 (P2X7R), which are promising for more specific visualization of the neuroimmune response, however research into these agents is still in early stages (102, 107).

There have been a handful of studies using TSPO PET after TBI. One early study using a first-generation tracer, ^11^C-(R)-PK11195, showed that after TBI, from 11 months up to 17 years, that TSPO PET remained persistently elevated in the thalami, putamen, occipital cortices, and posterior limb of the internal capsules, although not at the original injury site. They also found that the increase in TSPO PET uptake was correlated with worse cognitive outcomes, although not with initial severity of the injury nor the time since injury (108). A more recent pilot studying investigating TSPO PET using the ^11^C-DPA-713 tracer in a cohort of former American football players with TBI exposure, compared to age and sex matched controls, found that there was elevated TSPO in the supramarginal gyrus and right amygdala, along with atrophy in the right hippocampus. These changes were associated with varied performance on verbal learning and memory tests (109). Additional work by the same group showed that the individuals with TBI exposures had persistent glial activation in the bilateral hippocampus, bilateral parahippocampal cortices, bilateral supramarginal gyrus, left entorhinal cortex, and left temporal pole. Interestingly, they did not find a difference between the two groups in terms of in brain volume or neuropsychological performance (110).

While this is a relatively new area of active investigation, review of the clinical trial database on ClinicalTrials.gov shows 5 studies currently listed evaluating “TSPO + PET” for “traumatic brain injury.” Of these, 1 is completed with results, 2 are completed without results, and 2 are recruiting. The study that is completed with results (NCT01547780) set out to investigate if the TSPO PET tracer ^11^C-PBR28 was able to detect neuroinflammation in TBI in patients with acute and chronic TBI history. They found that levels of TSPO PET binding was highest in chronic TBI, but not in acute TBI. These results may support the use of TSPO PET to investigate chronic neuroinflammation after TBI. So far, the data that this group has generated has contributed to a publication detailing the levels of TSPO binding in the healthy control group (111). Of the two studies that have been completed without results, the first (NCT05183087) had the goal of studying TSPO PET using ^11^C-PBR28 as a biomarker for neuroinflammation in a group of military personnel with a history of repetitive low-level blast exposure. The second (NCT03482115) aimed to explore TSPO PET with the tracer ^11^C-DPA-714, in addition to other neuroimaging methods, in patients with traumatic or anoxic coma. While we are still in the early stages for the clinical study of TSPO PET for TBI, these studies highlight growing interest in TSPO PET as a tool for characterizing neuroinflammation and provide preliminary insight into its possible role in evaluating the chronic stages post TBI.

Interestingly, there have been a number of preclinical animal studies investigating the role of TSPO PET for evaluating neuroinflammation after TBI. One study evaluated neuroinflammation via TSPO PET using the tracer ^11^C-DPA-714 in a rat CCI model. PET imaging revealed significantly elevated tracer uptake in the injured brain region, with a high at day 6 post-injury and near normalization to baseline by day 28 (112). Another study using the tracer ^11^C-DPA-714 in a single mild CCI mouse model evaluated outcomes at both 7 and 21 days post injury and found that there was prolonged *in vivo* TSPO-related neuroinflammation in the ipsilateral cortex and hippocampus. Interestingly, there was no visible injury seen on MRI in the injured group in this study. These findings were confirmed with *ex vivo* immunohistochemistry which showed sustained microglial and astrocytic activation. Additionally, they found that the increase in TSPO PET uptake was correlated with cognitive and sensorimotor impairments (113). A third study investigated TSPO PET using the tracer ^11^C-DPA-714 in a CCI mouse model, and found that the TSPO PET uptake was correlated with the injury severity, increased astrogliosis, increased axonal injury, and increased activated microglia as evaluated by immunohistochemistry. However, one interesting finding was that in the mild TBI group, there was an increase in activated microglia as assessed by immunohistochemistry that was not seen on TSPO PET, highlighting the fact that TSPO PET may not be sensitive enough to detect changes early on in mild injuries (114). Overall, the preclinical data suggests that TSPO PET may be an effective *in vivo* biomarker for neuroinflammation, however further development of tracers and evaluation will be needed before this can be moved from the research arena and into clinical use.

In addition to TSPO, there has been research into the potential of using COX-2 PET tracers to study inflammation after TBI. COX-2 is an enzyme that is rapidly upregulated during inflammatory processes in the brain. It is not specific to brain injury, and is thought to play a role in numerous disease processes including TBI, but also AD, Parkinson’s disease (PD), Amyotrophic Lateral Sclerosis (ALS), epilepsy, stroke, and psychiatric disorders. There have been a handful of COX-2 PET radiotracers developed, but the ones most often used are ^11^C-MC1, ^11^C-MI, and ^18^F-MTP. COX-2 PET imaging is thought to have a few advantages over TSPO PET imaging for neuroinflammation, including faster and more transient upregulation, especially helpful in monitoring the acute phase of TBI (115). A recent paper was published studying the potential for the COX-2 specific PET tracer ^11^C-MC1 for evaluating neuroinflammation, like might be seen after TBI. In this study, they found that ^11^C-MC1 was able to detect low levels of COX-2 in healthy human brain, specific for the human isoform (116). These findings lay the groundwork for further analysis of this tracer after conditions like TBI which result in increased levels of neuroinflammation.

Preclinical studies using neuroinflammation PET markers, such as TSPO, corroborate clinical findings of persistent neuroinflammation after TBI and suggest that glial activation follows a spatial and temporal pattern that is dependent on TBI severity. The ability to correlate imaging with cellular markers in animal models helps interpret TSPO PET signal changes observed in humans, particularly when structural imaging is unremarkable. In addition to TSPO, the emerging COX-2 PET tracers offer a complementary approach, by targeting more transient inflammatory responses, with the potential to improve detection of acute phase specific neuroinflammation after TBI. PET imaging of neuroinflammation after TBI is an emerging area of research. Tracers targeting TSPO and COX-2 show promise for visualizing *in vivo* chronic neuroinflammation. Early clinical and preclinical studies suggest utility for tracking chronic neuroimmune responses, though further validation and development are needed. However, there are important limitations to the use of TSPO PET in TBI. One issue is the rs6971 polymorphism, which affects binding affinity for second-generation TSPO tracers, necessitating genotyping to ensure effective interpretation of PET signal across subjects. In addition to the issues with genetic variability, TSPO is a general marker of glial activation and is expressed by multiple cell types, which limits its cellular specificity and complicates interpretation of elevated TSPO signal. Finally, it is nearly impossible to tease out a beneficial inflammatory response from a detrimental prolonged inflammation using this tracer. These limitations highlight the need for more specific neuroinflammatory markers and improved imaging strategies to better characterize the complex immune responses following TBI.

### Emerging tracer PET

Finally, there are a handful of targets that have not been well studied in the human population after TBI but are still in development. These include neurotransmitters, such as gamma-aminobutyric acid (GABA) and dopamine, which are known to be disrupted after injury, as well as synaptic density markers.

GABA is the brain’s primary inhibitory neurotransmitter. It plays an important role in maintaining the balance of neuronal activity. The PET tracer ^11^C-flumazenil (FMZ) binds selectively to the benzodiazepine site of the GABA_A_ receptor and allows *in vivo* imaging of the brain’s main inhibitory regulator (117). There have only been a few studies evaluating these PET agents in TBI. Early studies found that relative to controls, individuals who had had diffuse axonal injury after TBI had decreased ^11^C-FMZ PET uptake in the bilateral medial frontal gyri, anterior cingulate, and thalamus. These regions are known to be associated with GABA_A_ receptor density, suggesting that these individuals have impaired GABA functioning after injury. They had also seen a correlation of reduced ^11^C-FMZ PET uptake and impaired cognitive performance, highlighting the role that these regions may play in detrimental changes after TBI (118). Another early study showed that in patients who had TBI with persistent symptoms, with normal MRIs, that there were concomitant decreases in uptake brain regions with^11^C-FMZ and ^15^O-labelled gas PET, highlighting the role for ^11^C-FMZ PET in evaluating subtle metabolic dysfunction in patients with normal structural imaging (119). An additional study showed that in a small population of retired boxers with TBI history, there was decreased ^11^C-FMZ PET uptake in the angular gyrus and temporal cortex, indicating GABA_A_ receptor deficits. Interestingly, they did not see any structural damage on MRI in this population (120). A more recent study used ^11^C-FMZ PET to assess patients with a history of chronic TBI, but normal structural MRIs. They found that in this population, relative to controls, there was decreased ^11^C-FMZ PET uptake binding in several thalamic nuclei, including the central, mediodorsal, anterior, and ventral regions. They also found that these changes in uptake were correlated with worse functional, cognitive, and emotional outcomes. Overall, these studies highlight the potential of ^11^C-FMZ PET to detect GABA_A_ receptor dysfunction after TBI, particularly in patients with normal structural imaging, and support its potential role as a sensitive marker of chronic neuronal impairment after TBI.

Dopamine plays an important role in reward, motivation, motor control, and cognitive function. Its dysregulation has been implicated in neuropsychiatric and neurodegenerative disorders. A number of dopamine PET agents have been developed, including ^11^C-*β*-CFT for the dopamine transporter, ^11^C-raclopride for D2 receptors, and ^11^C-PHNO for combined D2/D3 receptor binding (26). One study used ^11^C-PHNO PET in a cohort of 12 patients with moderate to severe TBI, half of whom were also suffering from post-traumatic depression. They found that patients with TBI history had decreased ^11^C-PHNO PET uptake in the caudate, relative to controls. Interestingly, those who were not also suffering from depression had increased ^11^C-PHNO PET uptake in the amygdala. The authors posit that this may be due to compensatory changes in this population (121). Another study found that after moderate to severe TBI, that there was decreased ^11^C-*β*-CFT PET uptake in the caudate, putamen, and ventral striatum and mildly increased ^11^C-raclopride PET uptake in the in the striatum relative to controls. These findings indicate reduced dopamine transporter binding, but increased D2 receptor binding, which is consistent with a hypodopaminergic state after TBI. Further, they found that genetic variants in the DAT and DRD2 genes modulated these effects. These results suggest that after TBI, there may be dysfunction in the dopamine system, which may be affected by genetic factors (122). Although research in this area remains limited, these findings support the presence of dopaminergic dysfunction after TBI and highlight the potential utility of dopamine PET imaging as in *in vivo* biomarker to track these changes.

Another target that that has not been well studied in TBI but has been studied in spinal cord injury and stroke is synaptic density tracers. The leading PET tracer for these studies is ^11^C-UCB-J, which binds to synaptic vesicle glycoprotein 2A (SV2A), a protein that is expressed in presynaptic terminals. ^11^C-UCB-J PET therefore provides a quantitative measure of synaptic integrity. In a study using the synaptic density tracer ^11^C-UCB-J PET after stroke, they found that patients with stroke had a decrease in ^11^C-UCB-J PET uptake in the ischemic core and peri-ischemic areas over time, consistent with loss of neuronal tissue (123). These findings, while not directly related to TBI, highlight the ways in which this marker may be used as an *in vivo* method to track synaptic density after TBI. Emerging PET tracers targeting GABA, dopamine, and synaptic density offer promising new methods for detecting neuronal dysfunction after TBI, particularly in patients with normal structural imaging. Preliminary studies suggest that PET tracers targeting GABA_A_ receptors (^11^C-FMZ), dopamine signaling (^11^C-*β*-CFT, ^11^C-raclopride, ^11^C-PHNO), and synaptic density (^11^C-UCB-J) may offer unique markers of neuronal dysfunction after TBI (Table 1). However, there are significant limitations to the emerging tracers discussed here. Emerging tracers targeting GABA, dopamine, and synaptic density offer mechanistic insights, but their potential use in TBI is currently limited by sparse preclinical validation, limited clinical studies, and challenges in distinguishing TBI-related signal changes from baseline variability or comorbid neuropsychiatric conditions.

**Table 1:** Summary of the reviewed diagnostic PET tracers, their biological targets, research focus in TBI, and current stage of development.

Target	Tracer(s)	Research questions in TBI	Stage
Glucose	^18^F-FDG	Hypo/hypermetabolism	Preclinical/Clinical
Amyloid	^11^C-PiB, ^18^F-florbetapir, ^18^F-florbetaben, ^18^F-flutemetamol	Amyloid accumulation, TBI vs AD	Preclinical/Clinical
Tau	First Generation (^18^F-flortaucipir, ^11^C-PBB3, ^18^F-THK family) Second Generation (^18^F-florquinitau)	Post TBI tauopathy, CTE vs AD	Preclinical/Clinical
Amyloid + Tau	^18^F-FDDNP	CTE diagnosis	Preclinical/Clinical
TSPO	First Generation (^11^C-(R)-PK11195) Second Generation (^11^C-PBR28, ^11^C-DPA-714)	Chronic inflammation	Preclinical/Clinical
COX-2	^11^C-MC1, ^11^C-MI,^18^F-MTP	Acute Inflammation	Preclinical
GABA	^11^C-flumazenil	Thalamic and frontal GABAergic dysfunction	Preclinical/Clinical
Dopamine	^11^C-raclopride, ^11^C-PHNO, ^11^C-β-CFT	Post TBI hypodopaminergic state, depression	Preclinical/Clinical
SV2A	^11^C-UCB-J	Decreased synaptic density post TBI	Preclinical

## Therapeutic implications of PET tracers in TBI

PET imaging has traditionally been employed as a diagnostic tool to visualize molecular and cellular pathology; however, the development of PET imaging biomarkers has the potential to transform treatment strategies in TBI by enabling *in vivo* visualization of specific pathophysiological processes, guiding treatment decisions. The field of nuclear medicine has recently developed the term “theranostics” to describe the integrated use of diagnostic imaging paired with targeted therapy. This term has really taken off with the advent of PSMA targeted PET imaging paired with PSMA targeted radioligand therapy. Since the advent of this term, theranostics has most commonly been applied to oncology pairs such as these. However, there has been a push to use this term more broadly across disciplines (124). Building on this paradigm, is the idea that the term “neurotheranostics,” would more fully encompass the idea of using paired PET neuroimaging with targeted treatment (125).

This concept is perhaps best illustrated in the context of AD. The amyloid cascade hypothesis, first proposed in the early 1990s, is the idea that Aβ is an initiating event in AD pathology. This idea was supported by early findings tying strong familial cases with mutations such as APP and PSEN1. However, more recent evidence has raised questions about whether this amyloid deposition is actually causal of clinical disease (126). It seems likely that there is complex temporal relationship between amyloid deposition, tau pathology, and neurodegeneration that is occurring. Even so, anti-amyloid therapies have shown promise in decreasing amyloid burden, and there is the hope that this translates in a modification of the disease course. In those treated early on, there is the hope that there can be delayed clinical disease onset (127). In those treated with significant amyloid burden, there is the hope that treatment can slow progression of disease (128, 129). Since the FDA granted accelerated approval to lecanemab in January 2023, followed by full approval in July 2023, and approved donanemab in July 2024, and with the EMA approval of lecanemab in April 2025, there has been growing need to evaluate who is an appropriate candidate for these anti-amyloid therapies. There has been a subsequent clinical increase in the usage of amyloid PET to evaluate amyloid burden before the clinical decision is made to treat patients with ant-amyloid therapies. This combination of paired PET imaging and therapeutic agent exemplifies the potential power of neurotheranostics.

Drawing from this illustrative case of Alzheimer’s disease, there is currently untapped potential for personalized treatment strategies in TBI. Throughout this review, we have discussed several classes of PET tracers relevant to TBI, including those targeting glucose metabolism (^18^F-FDG), pathological protein aggregates such as amyloid (^11^C-PiB, ^18^F-florbetapir, ^18^F-florbetaben, ^18^F-flutemetamol) and tau (^18^F-flortaucipir, ^18^F-MK-6240), neuroinflammation (TSPO ligands such as ^11^C-PBR28 and ^11^C-DPA-714; COX-2 ligands such as ^11^C-MC1), synaptic density (^11^C-UCB-J), and neurotransmitter systems including GABAergic signaling (^11^C-flumazenil) and dopaminergic function (^11^C-PHNO, ^11^C-raclopride, ^11^C-*β*-CFT). Leveraging these molecular imaging tools, neurotheranostics offers a pathway toward personalized TBI treatment, where interventions can be selected and monitored based on the actual underlying pathophysiological processes in each individual.

Here, we will briefly review the major classes of PET biomarkers discussed in this review and explore their potential roles as neurotheranostic tools when paired with existing or emerging therapies for TBI. While most therapeutic strategies remain in early development or are adapted from other disease contexts, such as AD, we summarize the current state of research investigating their application in TBI and highlight opportunities for future therapeutic pairing and clinical translation.

### Amyloid therapies

The use of amyloid PET has facilitated the development of targeted therapies which reduce amyloid in the brain. Most anti-amyloid treatments have been developed in the context of AD, as discussed previously. While a few preclinical studies have investigated anti-amyloid treatments in animal models of TBI, a review of ClinicalTrials.gov reveals no currently registered studies using “anti-amyloid” agents for “traumatic brain injury.” One preclinical study a CCI rat model of TBI infused an anti-amyloid precursor protein (APP) antibody directly into the perilesional cortex, resulting in reduced apoptosis, smaller lesion volume, and improved cognitive performance (130). However, there are no results for studies evaluating the agents lecanemab or donanemab in TBI. The lack of clinical research into anti-amyloid therapies for TBI is likely multifactorial. First, the current literature does not consistently demonstrate that amyloid accumulation plays a central role in TBI pathophysiology, as it does in AD. More robust, longitudinal preclinical animal model studies would be needed to justify clinical trials. Second, the patient population with TBI is heterogeneous, and identifying individuals with significant amyloid pathology after injury remains challenging, limiting the feasibility of selecting appropriate candidates for anti-amyloid interventions. Further research into amyloid accumulation after TBI is warranted and may eventually support the development of a neurotheranostic pair.

### Tau therapies

Next, we discussed tau pathology in the brain after TBI. Tau accumulation, particularly in the chronic phase of TBI, has been associated with neurodegeneration, cognitive decline, and an elevated risk of developing long-term tauopathies. Evidence for increased tau deposition after TBI was more consistent and compelling than that for amyloid. Anti-tau therapies exist across multiple modalities, including small molecules such as sodium selenate (131), AAV vectors such as AADvac1 (132–134), and monoclonal antibodies such as semorinemab (135, 136).

Recent experimental work has begun to evaluate these anti-tau therapies in animal models of TBI, investigating their mechanisms of action and providing evidence for their translational potential. In one study, they used a mouse model of multimodal TBI, the Jet-Flow Overpressure Model (137), and they found that tau acetylation at lysine 174, a process that impairs degradation and promotes tau accumulation (138), increases after TBI and drives neurodegeneration. Methods of treatment that target anti-tau acetylation, such as inhibiting GAPDH nitrosylation, blocking p300/CBP, or activating Sirtuin1 have showed reduced brain atrophy, neuronal loss, and cognitive deficits in their model (139). In another study, they utilized a CCI TBI model in a PS19 tauopathy mouse model. They tested two monoclonal antibodies specifically targeting acetylated tau at lysine 174 (ac-tauK174). They found that treatment with anti-ac-tauK174 prevented TBI-induced neurodegeneration, preserved memory function, and reversed glial transcriptomic changes (140). Another study targeted tau a different way, focusing on phosphorylated tau (p-tau). In a repeated CCI injury model meant to mimic CTE, they used an AAV vector coding for an anti-p-tau antibody, and found that in their model, they were able to decrease p-tau in the brain after repeated TBI using this therapy (141). While monoclonal antibodies against tau have been well studied in AD (135, 142), investigation into monoclonal antibodies against tau in TBI are still in early phases (143). One study showed that treatment with a cis p-tau specific monoclonal antibody, clone #113, prevented tauopathy development, rescued neuronal and behavioral deficits, and reduced brain atrophy in a repeated CCI mouse injury model (144).

While there have been several preclinical studies investigating anti-tau treatments in animal models of TBI, a review of ClinicalTrials.gov reveals no currently registered studies using “anti-tau” agents for “traumatic brain injury.” There are two clinical trials listed which plan to study the anti-tau agent MK-2214 (NCT05466422, NCT07033494) in AD, but they are not yet recruiting.

### Neuroinflammation therapies

Neuroinflammation is a central component of secondary injury following traumatic brain injury. In the previous section we primarily discussed TSPO PET imaging as a valuable tool visualizing and quantifying activation of the neuroimmune system *in vivo.* We also discussed new research being done evaluating COX-2 as a marker for inflammation. There has been significant research studying therapies targeting neuroinflammation after TBI, most of which is outside the scope of this review. Here, we will focus on research being done investigating agents that could be used as a neurotheranostic pair, focusing particularly those agents targeting TSPO and COX-2.

There are a number of small molecules which have been used as TSPO ligands, such as PK 11195, Ro5-4864, and etifoxine (145). Some of these have been utilized as PET ligands, while others have been utilized primarily as ligands in research biochemical assays and preclinical research. While clinical research has been limited, a number of preclinical studies have evaluated the use of etifoxine as a therapy after TBI. One group found that in a CCI rat model, that treatment with etifoxine after TBI improved sensorimotor function deficits, as well as led to a reduction in pro-inflammatory cytokines, reduced macrophages and glial activation, and reduced neuronal degeneration (146). Another group found that in a rat CCI model, treatment with etifoxine after TBI induced a significant reduction in lesion volume in a dose-dependent manner, improved neurological outcomes at 4 weeks, enhanced neuronal survival, and reduced apoptosis (147). In a separate study, the same group also reported that etifoxine significantly improved cognitive function as evidenced by faster recovery in Morris water maze testing (148).

In addition to TSPO, there is growing interest in COX-2 as a target for developing PET tracers to image neuroinflammation in the brain following TBI. There have been numerous animal studies showing preliminary evidence that targeting COX-2 may be a valuable target to decrease neuroinflammation and neuronal cell death and to improve outcomes after TBI (149). Interestingly, a retrospective cohort study found that early treatment with the COX-2 inhibitor celecoxib within 5 days of traumatic brain injury was associated with significantly improved 1-year survival and reduced rates of complications (150). However, prospective studies in humans are needed to further determine the clinical utility of this treatment approach post TBI.

### Emerging therapies

Finally, in the emerging tracers PET section, we discussed PET agents targeting GABA_A_ receptors (^11^C-FMZ), dopamine signaling (^11^C-*β*-CFT, ^11^C-raclopride, ^11^C-PHNO), and synaptic density (^11^C-UCB-J). Research investigating these agents is still in preliminary stage, but has the potential to be translated into promising clinical work.

The GABAergic system in the brain is complex; it is thought that enhancing GABA_A_ receptor activity acutely after TBI may reduce excitotoxic injury, while later, inhibition of excessive GABAergic tone may support recovery of cognitive function by promoting neural plasticity (151). One group found that enhancing GABA_A_ receptor activity with diazepam during the acute post-injury period reduced mortality and improved cognitive performance in an LFP rat model of TBI (152), while another group found that in a CCI rat model, that treatment with flumazenil significantly improved learning and memory in a dose-dependent manner (153). Together, these findings highlight the time-sensitive and dynamic role of GABAergic signaling in TBI recovery.

Another neurotransmitter system implicated in TBI pathophysiology is dopamine, which modulates reward, arousal, and cognitive function. After TBI, it is thought that a hypodopaminergic state is induced. Studies have investigated whether treatment with agents that interact with the dopaminergic system might be beneficial after TBI, or related conditions such as stroke (154). After TBI, treatment with amantadine, a dopamine reuptake inhibitor and an NMDA receptor antagonist, has been shown to reduce irritability and aggression in individuals with a history of chronic TBI of greater than 6 months (155). Additional research has been done in the related field of strokes. One study found that the use of levodopa, a dopamine precursor, and methylphenidate, a dopamine and norepinephrine reuptake inhibitor, resulted in improvements in activities of daily living and stroke severity over 6 months post-stroke (156), while another study found that rotigotine, a dopamine agonist, improved visual search and selective attention post stroke (157). However, studies investigating dopaminergic agents after TBI have been limited due to lack of clear pathology being targeted as well as the significant side effects that these medications can have, given the wide role of dopamine in the brain.

Finally, while not currently well studied in TBI, synaptic density is another measure that PET tracers such as ^11^C-UCB-J can assess. Therapies that aim to stimulate synaptic growth, such as using agents targeting the RhoA-ROCK signaling pathway (158) or treatment with agents like stem cell factor and granulocyte colony-stimulating factor may prove beneficial (159).

These are promising emerging therapeutic targets for TBI, but current evidence is still preliminary and further research is needed to validate their clinical relevance and guide effective intervention strategies (Table 2). Pairing interventions such as these with PET tracers would offer a powerful neurotheranostic approach, enabling real-time monitoring of pathophysiology and treatment response.

**Table 2:** Summary of the reviewed potential neurotheranostic pairs in TBI, diagnostic targets, mechanisms of action, and development stage.

Diagnostic target	Therapeutic pair	Mechanism	Stage
Amyloid	Monoclonal antibodies (lecanemab, donanemab) Anti-amyloid precursor protein antibody	Clears amyloid	Preclinical
Tau	Small molecules (sodium selenate) AAV vectors (AADvac1) Monoclonal antibodies (semorinemab, anti-ac-tauK174)	Blocks tau accumulation	Preclinical
TSPO	Small molecules (etifoxine)	Reduces microglial activation	Preclinical
COX-2	Nonsteroidal anti-inflammatory drugs (celecoxib)	Blocks neuroinflammation	Observational
GABA	Benzodiazepines (diazepam) Benzodiazepine antagonists (flumazenil)	Modulates inhibitory tone	Preclinical
Dopamine	Dopamine reuptake inhibitors (amantadine) Dopamine precursors (levodopa) Dopamine agonists (rotigotine)	Increases synaptic dopamine	Preclinical
SV2A	Cell signaling modulators (RhoA-ROCK inhibitors) Growth factors (stem cell factor, granulocyte colony-stimulating factor)	Promotes synaptic repair	Preclinical

### Toward clinical translation: a roadmap for neurotheranostics in TBI

To realize the potential of neurotheranostics in TBI, several discrete steps must be taken to move from preclinical biomarker discovery into clinical application. The first step is to validate PET tracers in well-characterized TBI populations. For example, tau PET imaging agents show potential for identifying TBI-related tau deposition in the brain. However, there is a lack of robust, replicated, large-scale studies with postmortem confirmation. Further characterization and validation of leading biomarker contenders is essential for advancing a neurotheranostic approach in TBI.

Once validated tracers have been established, a paired therapy must demonstrate clinical efficacy in the TBI patient population. Continuing with the example of tau, numerous anti-tau therapies have been developed, though primarily for Alzheimer’s disease. Preclinical models must be established, and these agents must be thoroughly tested in those models before progressing to clinical trials in humans with TBI.

Finally, translation from bench to bedside will require multidisciplinary collaboration between academia, clinicians, industry, and regulators. Clear identification of patients likely to benefit from the intervention is needed. Treatment timepoints must be selected to maximize potential benefit, and there must be evidence that PET biomarkers improve following treatment and that these improvements correspond to functional improvement. Once this is shown, regulatory approval must be pursued, demonstrating that the neurotheranostic pair helps identify responsive patients and that treatment leads to clinical improvement.

Although these steps remain aspirational, they offer a conceptual framework for how neurotheranostics could be implemented in the context of TBI.

## Conclusion

PET imaging has emerged as a critical tool in advancing our understanding of the molecular and cellular mechanisms underlying traumatic brain injury. Through tracers targeting glucose metabolism, amyloid, tau, neuroinflammation, synaptic integrity, and neurotransmitter systems, PET enables noninvasive*, in vivo* assessment of complex and dynamic pathological processes. However, if we aim to realize a neurotheranostic approach for TBI, several vital steps must be taken to translate PET-guided strategies from experimental tools into clinically actionable interventions.

First, to realize the full potential of this approach, it would be wise to focus future efforts on the most promising translational applications. To do this, identifying the most promising diagnostic PET tracers and therapeutic pairs is vital, with an emphasis on those combinations that have the highest potential for successful clinical translation. This approach can help prioritize research investments and ensure that high-yield targets receive the support needed to make the challenging transition from bench to bedside.

Of the various diagnostic PET agents reviewed here, both amyloid and tau imaging stand out as the most immediate candidates for translational application, due to their established use in AD and the parallel development of targeted therapies. Diagnostic amyloid PET is already being used in AD in conjunction with anti-amyloid agents such as lecanemab and donanemab, meaning that much of the necessary infrastructure, including tracer production, integration into clinical workflows, and regulatory precedent, has already been established. However, the utility of amyloid PET and the potential use of a paired anti-amyloid treatment agent in TBI is not clear from the current literature. Studies investigating amyloid deposition in TBI have produced inconsistent results, complicated by variations in age, injury mechanism, post-TBI time course, and comorbid factors. These inconsistent findings raise the question of whether amyloid plays a central pathological role in TBI or if it is merely a secondary effect of other more primary mechanisms. Further research is needed to evaluate amyloid deposition in TBI before applying amyloid PET and therapeutic pairs for treatment.

On the other hand, diagnostic tau PET is arguably the most promising avenue for a neurotheranostic approach for TBI. Tau pathology appears more consistently associated with injury severity, especially in the case of patients with CTE. Anti-tau therapies, including monoclonal antibodies, AAV vectors, and small molecules, are currently being studied, with some already in clinical trials for Alzheimer’s disease, and could potentially be adapted for TBI studies. However, similar issues affect tau as they do amyloid, and given the heterogeneity of the disease process, more studies evaluating the role that tau plays and whether tau PET is useful in human TBI populations will be necessary before moving into tau-related treatment options.

PET imaging focused on neuroinflammation, using tracers such as TSPO or COX-2, may ultimately prove to be more biologically relevant to TBI, given the known central role that neuroinflammatory processes like microglial activation and astrogliosis play in secondary injury. However, both diagnostic PET agents and therapeutic developments in this space are still in the early stages. In addition, current tracers like TSPO suffer from known limitations, such as genetic variability in binding and off-target effects, which may hinder their ability to be translated to larger populations. Given the earlier stage of research that neuroinflammation PET is currently in, and the need for further refinement in tracer development, a neurotheranostic approach targeting neuroinflammation may follow a slower path to clinical integration, although it may ultimately prove to be the most useful.

Second, a major challenge for PET research is the heterogeneity of TBI, including variation in injury severity, type, timing, comorbid factors, post-injury progression, and the occurrence of multiple injuries. This complexity stands in contrast to the more uniform trajectory of Alzheimer’s disease. As a result, patient selection and imaging interpretation in TBI can be difficult. First, patients must be accurately classified into appropriate subgroups, a process that may benefit from emerging guidelines. Second, tracer selection must align with the injury subtype and timing, as some tracers may be better suited for chronic or repeated TBI, while others may be more informative in the acute setting. Third, continued tracer development and validation, including postmortem correlation, are essential to ensure that PET signal reflects true pathology and can reliably inform diagnosis and treatment planning.

While the translation of PET imaging into clinical care for TBI remains aspirational, it is increasingly feasible. Tau and amyloid represent the most developed tracer-therapy pairings, with potential for relatively short-term cross-application from Alzheimer’s disease. Neuroinflammation PET may offer greater biological relevance for TBI but will require a longer path to therapeutic pairing. Across all targets, patient stratification, study design, tracer development, and confirmation of tracer specificity will be essential. Addressing these challenges through coordinated research efforts will help establish PET-guided neurotheranostics as a viable method for personalizing treatment in TBI.
